# Influence of rhizobacterial volatiles on the root system architecture and the production and allocation of biomass in the model grass *Brachypodium distachyon* (L.) P. Beauv.

**DOI:** 10.1186/s12870-015-0585-3

**Published:** 2015-08-12

**Authors:** Pierre Delaplace, Benjamin M. Delory, Caroline Baudson, Magdalena Mendaluk-Saunier de Cazenave, Stijn Spaepen, Sébastien Varin, Yves Brostaux, Patrick du Jardin

**Affiliations:** University of Liège, Gembloux Agro-Bio Tech, Plant Biology, Passage des Déportés 2, 5030 Gembloux, Belgium; Department of Plant Microbe Interactions, Max Planck Institute for Plant Breeding Research, Carl-von-Linné-Weg 10, 50829 Köln, Germany; University of Liège, Gembloux Agro-Bio Tech, Applied Statistics, Computer Science and Modeling, Passage des Déportés 2, 5030 Gembloux, Belgium

## Abstract

**Background:**

Plant growth-promoting rhizobacteria are increasingly being seen as a way of complementing conventional inputs in agricultural systems. The effects on their host plants are diverse and include volatile-mediated growth enhancement. This study sought to assess the effects of bacterial volatiles on the biomass production and root system architecture of the model grass *Brachypodium distachyon* (L.) Beauv.

**Results:**

An *in vitro* experiment allowing plant-bacteria interaction throughout the gaseous phase without any physical contact was used to screen 19 bacterial strains for their growth-promotion ability over a 10-day co-cultivation period. Five groups of bacteria were defined and characterised based on their combined influence on biomass production and root system architecture. The observed effects ranged from unchanged to greatly increased biomass production coupled with increased root length and branching. Primary root length was increased only by the volatile compounds emitted by *Enterobacter cloacae* JM22 and *Bacillus pumilus* T4. Overall, the most significant results were obtained with *Bacillus subtilis* GB03, which induced an 81 % increase in total biomass, as well as enhancing total root length, total secondary root length and total adventitious root length by 88.5, 201.5 and 474.5 %, respectively.

**Conclusions:**

This study is the first report on bacterial volatile-mediated growth promotion of a grass plant. Contrasting modulations of biomass production coupled with changes in root system architecture were observed. Most of the strains that increased total plant biomass also modulated adventitious root growth. Under our screening conditions, total biomass production was strongly correlated with the length and branching of the root system components, except for primary root length. An analysis of the emission kinetics of the bacterial volatile compounds is being undertaken and should lead to the identification of the compounds responsible for the observed growth-promotion effects. Within the context of the inherent characteristics of our *in vitro* system, this paper identifies the next critical experimental steps and discusses them from both a fundamental and an applied perspective.

**Electronic supplementary material:**

The online version of this article (doi:10.1186/s12870-015-0585-3) contains supplementary material, which is available to authorized users.

## Background

Within the crop environment, both rhizospheric (underground) and phyllospheric (aboveground) bacteria greatly influence plant growth [1–3]. Free-living, biofilm-forming and root-colonizing rhizobacteria have therefore been considered as possible inoculants for increasing plant productivity and improving nutrient-use efficiency [4, 5].

Plant growth-promoting rhizobacteria (PGPR) can have complex effects on their host plants. The underlying mechanisms include; (1) root system architecture (RSA) modulation and increased shoot growth, mediated particularly by indole-3-acetic acid, cytokinins, gibberellins, salicylic acid, ethylene and brassinosteroids; (2) phosphate solubilisation; (3) free nitrogen fixation; (4) suppression of harmful microorganisms; (5) induced systemic resistance; and (6) induced systemic tolerance of abiotic constraints [1–4, 6, 7].

Among these interaction mechanisms, the emission of bacterial volatile organic compounds (VOCs) has been shown to promote plant growth [6] and VOC-mediated plant growth modulation is now widely considered to be an important mechanism [8]. Apart from inorganic molecules such as CO_2_, CO, H_2_, N_2_, N_2_O, NO, NO_2_, NH_3_, H_2_S and HCN, microorganisms are able to emit VOCs [5, 9–11]. These include acids, alcohols, ketones, aldehydes, esters, terpenoids, aromatic, nitrogenous and sulphurous compounds, and ethylene [7, 12, 13]. Among these compounds, although 300 candidate molecules have been identified to date, very few have been unequivocally identified as being responsible for the observed change in plant growth [8, 9], mainly because bacterial volatiles can act as individual compounds or in mixtures [13].

Bacterial volatile exposure can lead to an increase in plant biomass (up to sixfold) or to plant death after 21 days of the exposure of *Arabidopsis thaliana* (L.) Heynh to bacterial volatiles [8, 11]. In general, the positive effects of bacterial VOCs on plant growth have been less frequently documented than the negative ones [8, 14]. On the positive side, eight bacterial volatiles (2,3-butanediol, 3-hydroxy-2-butanone, 2-pentylfuran, N,N-dimethyl-hexadecanamine, CO_2_, 13-tetradecadien-1-ol, 2-butanone and 2-methyl-n-1-tridecene) have been shown to promote plant growth [6, 8, 15–19]. Short-term growth-promotion effects observed on the model plant, *A. thaliana*, exposed to *Bacillus subtilis* GB03 volatiles include: (1) modulations of cytokinin [6], ethylene [20, 21], auxin, salicylic acid, brassinosteroids, gibberellins [4], abscisic acid and jasmonic acid [21] signalling pathways; (2) higher photosynthetic capacity, chloroplast number, chlorophyll content, starch accumulation and iron uptake [22]; (3) increased tolerance of osmotic, salt and drought stress through the accumulation of choline and glycine betaine in plant tissues [23, 24]; (4) reduced severity of disease symptoms; (5) reduced sensitivity to reactive oxygen species [25]; and (6) increased resistance against pathogens [13]. Similar long-term effects have been described [26] and for other plant species, such as *Nicotiana benthamiana* Karel Domin [27, 28] and *Agrostis stolonifera* L. [11, 29].

On the other hand, neutral or negative effects of rhizobacterial volatiles have been noted on plants, fungi and pathogenic bacteria [14, 30]. Hydrogen cyanide, which is produced by a small number of bacterial species including *Pseudomonas* [10] and *Chromobacterium* species, might be responsible for their negative impact on wheat. Complementarily, the negative effects of *Serratia* species on *A. thaliana* have been ascribed to dimethyl disulphide, β-phenyl-ethanol and the inorganic volatile NH_3_ [8, 13, 18, 31].

Despite these studies, several questions remain unanswered and need to be addressed. So far, only two studies on the impact of rhizobacterial volatiles on grass growth have been published. The observed effects were negative [32] or non-significant [30]. Until now, therefore, no clear growth-promotion effect of bacterial volatiles on *Poaceae* has been demonstrated. The root system development of members of the grass family differs in overall architecture and in the anatomy of individual roots [33], which could result in volatile effects that are different from those known with *A. thaliana* (Monocots *vs* Dicots, respectively).

With regard to RSA measurements, most *in vitro* studies use horizontal Petri dishes in which roots are grown in the agar plate, thus limiting their exposure to water-soluble volatile compounds. In addition, few studies have sought to characterise microbial volatile-mediated RSA modification using a dedicated experimental set-up [7, 13, 18, 34]. The few results that do exist suggest that bacterial VOCs are able to modulate root system morphogenic processes and that these RSA modifications could be related to biomass production [7, 13].

The aim of this study was to investigate the impact of bacterial volatile compounds on the biomass production and RSA of *Brachypodium distachyon* (L.) Beauv. (line Bd21), based on a 10-day *in vitro* co-cultivation. The genus *Brachypodium* is phylogenetically close to the temperate cereal genera *Triticum, Hordeum* and *Avena* in the subfamily Pooideae [35, 36] and it is now considered to be a promising model genus for studying root system development in cereals and the impact on plant yield [37, 38]. In this study we sought to answer the following questions: What are the main plantlet phenotypes induced by bacterial volatiles? Based on the biomass production and RSA results in our screening system, which strains have the most significant effect? How do the observed effects differ from those reported for dicotyledonous plants such as *A. thaliana*? The results are discussed within the context of the potential and limits of the *in vitro* system used in the study.

## Results

### Characteristics of the *in vitro* co-cultivation system

**Fig. 1:**
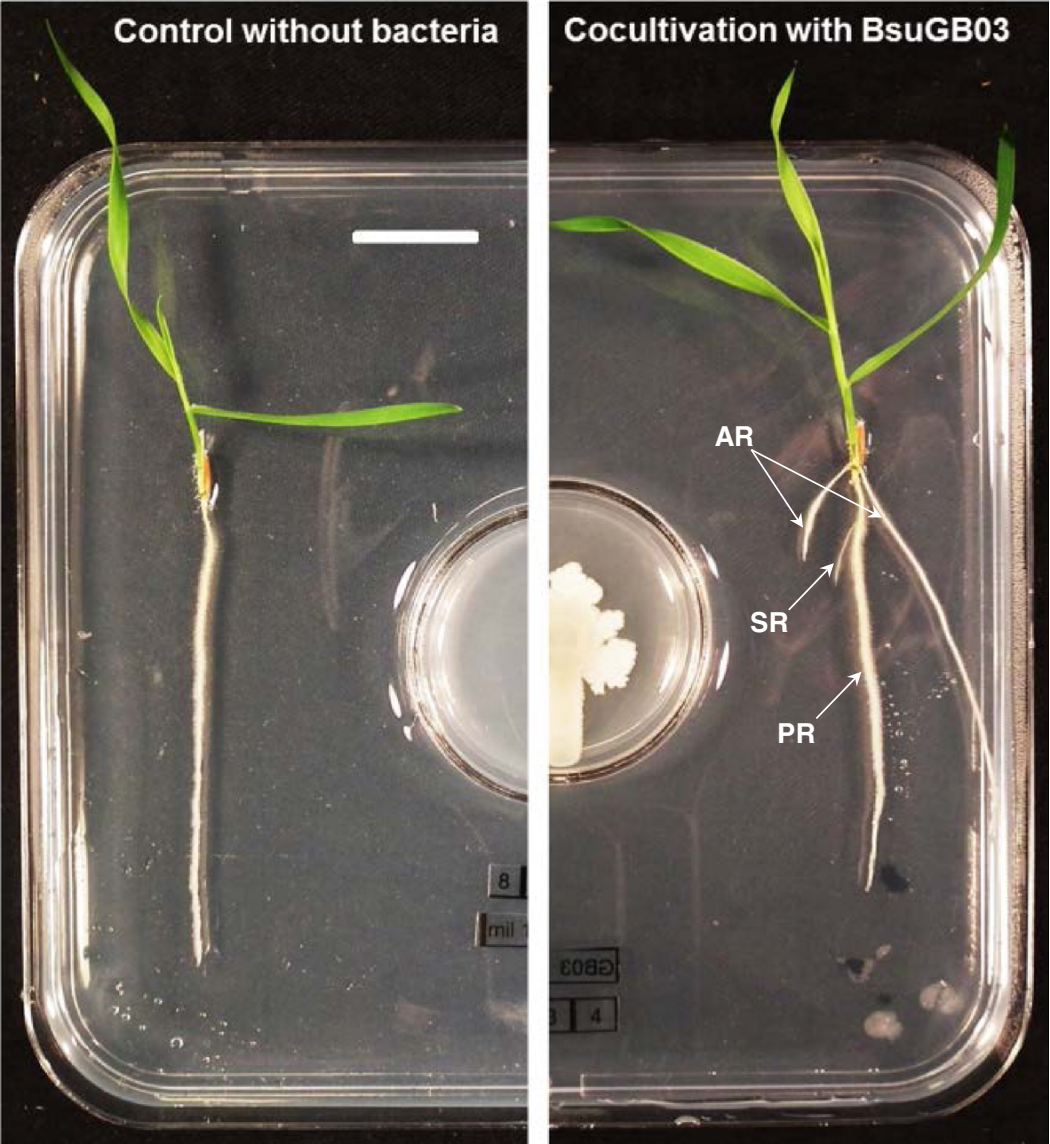
*In vitro* co-cultivation system. *B. distachyon* Bd21 plantlets were photographed after 10 days of near-vertical growth without (left) or with (right) exposure to BsuGB03 volatiles. The bacterial compartment contains a Farag *et al.* [15] medium and the plant compartment contains a Hoagland agar plate. Both growing media are physically separated, which limits plant-bacteria interactions to the exchange of volatiles. The scale bar is 1.75 cm long. The arrows point the adventitious roots (AR), the secondary roots (SR) and the primary root (PR) locations

In order to expose *B. distachyon* Bd21 plantlets to bacterial volatile compounds and assess their effects on biomass production while measuring RSA parameters, a near-vertical co-cultivation system was set up (Fig. 1). The bacterial growth media was based on the work of [15] and its composition was a compromise between a minimal medium and a nutrient one. The plants were grown on an agar plate containing Hoagland’s medium, which was physically separated from the bacteria, but shared the same atmosphere. The plantlets could be maintained in this system at 22 °C for up to 10 days. The leaves and roots grew on top of the agar plate and were therefore exposed to bacterial volatiles, whatever their polarity or solubility in the agar. Three kinds of roots were potentially produced by the plantlets: a primary root (PR), secondary roots (SR, branching from the PR) and adventitious roots (AR, Fig. 1). These three types of roots correspond to the ‘primary seminal axile root’, the ‘branch roots’ and the ‘coleoptile nodal roots’ defined by [39]. This experimental set-up did not induce any gradient effects because all the plantlets were positioned at the same distance from the source of the volatile compounds.

### Principal component analysis (PCA) and strain clustering

**Fig. 2:**
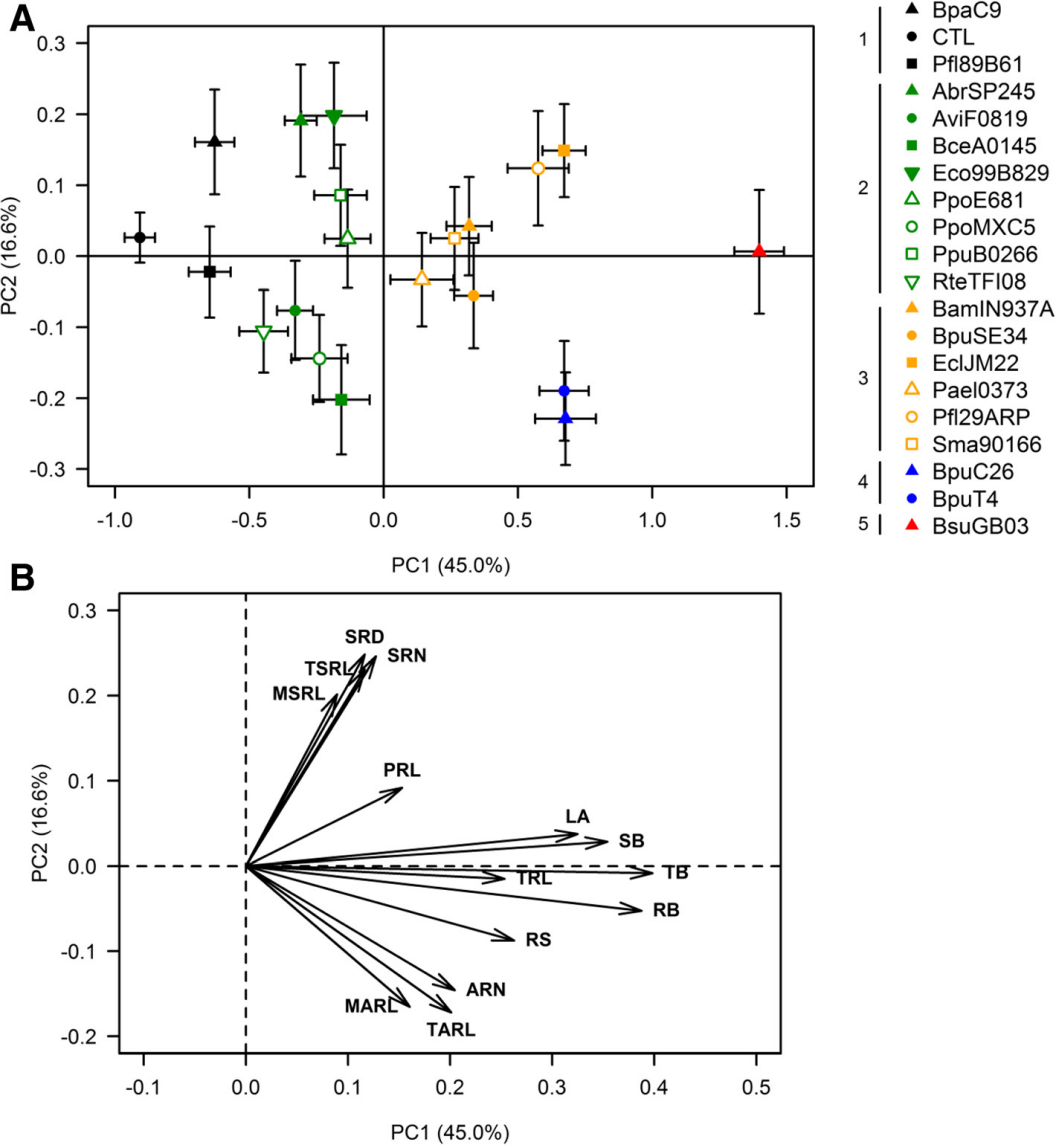
PCA based on individual weighted and reduced data (**a**) and correlation circle between the 14 measured variables and the two first components of the PCA (**b**). Presented values are means of 64 or 128 biological replicates +/− standard error of the mean for each strain and the control, respectively. Each of the five clusters defined by the hierarchical clustering processing is presented in a different colour: cluster 1, (including the control) black; 2, green; 3, yellow; 4, blue; and 5, red. PC 1 is correlated mainly with the biomass production of the plantlets exposed to the bacterial volatile compounds, whereas PC2 is related to RSA modulation. The proportion of the total variance explained by the two first axes is 61.6 %

Fourteen variables were measured on the *B. distachyon* plantlets after 10 days of volatile compound-based interactions with each of the 19 bacterial strains (Figs. 4 and 5, Additional file 1: Figure S1). In order to group the strains in terms of their growth-modulation ability, a PCA was performed on the dataset based on weighted and reduced variables (Fig. 2). This processing enabled us to assign the same weight for biomass- or RSA-related variable classes. Within each class, each variable had the same weight, irrespective of its order of magnitude. The 14 principal components (PCs) were then used as input variables to cluster the strains based on the Euclidian distance and the Ward algorithm.

Axes 1 and 2 account for 61.6 % of the total variance. Axis 1 is positively correlated with the growth-promotion ability of the strains, namely the total biomass (TB), root biomass (RB), shoot biomass (SB), leaf area (LA) and total root length (TRL) values (Fig. 2b). In contrast, axis 2 is related to RSA modulation and is positively correlated with secondary root growth (secondary root number (SRN), total secondary root length (TSRL), mean secondary root length (MSRL) and secondary root density (number of secondary roots per cm of primary root, SRD)) and negatively correlated with adventitious root growth (adventitious root number (ARN), total adventitious root length (TARL) and mean adventitious root length (MARL)).

**Fig. 3:**
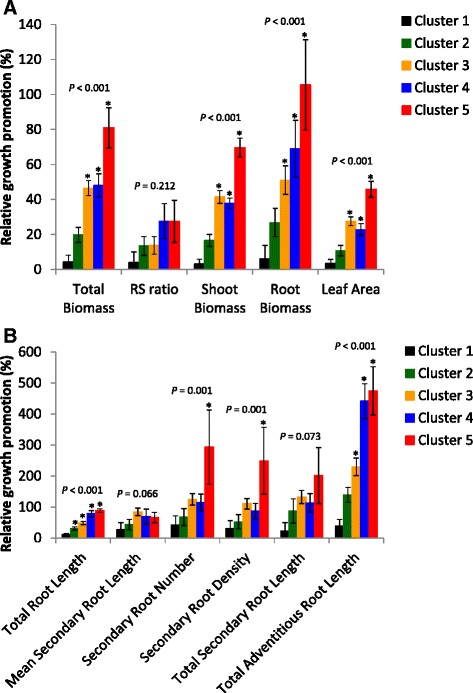
Relative growth promotion effects (%) on biomass (**a**) and RSA (**b**) variables. Each presented value is the mean of the relative differences between the replicates of the strains within a given cluster and the control without bacteria +/− standard error of the mean. RSA parameters with the five highest correlation coefficients to PC1 and PC2 are presented. The *P*-values are displayed on the graphs. Significant changes compared with the control without bacteria are marked with an asterisk (*)

Based on the PC values, the clustering algorithm allowed us to define five clusters of strains that induced consistent changes in the plantlet phenotypes (Fig. 3). Cluster 1 contained strains that did not affect plant phenotype significantly compared with the control. Only three Cluster 2 strains slightly increased biomass production, but the overall effect was not significant. The Cluster 2 strains effects on the root branching process were variable. Strains in clusters 3 and 4 greatly increased biomass production, but had variable effects on RSA. Cluster 5 contained only one strain, which had the greatest growth-promotion ability (Fig. 3a).

### Cluster composition (Fig. 2a)

Cluster 1 contained BpaC9 and Pfl89B61 in addition to the control (growth medium without bacteria). Cluster 2 had eight strains belonging to seven species and grouped into two sub-groups: (1) AbrSP245, Eco99B829, PpoE681, PpuB0266 with positive PC2 values; and (2) AviF0819, BceA0145, PpoMXC5 and RteTFI08 with negative PC2 values. Among the strains in cluster 3, most of them (BamIN937A, BpuSE34, EclJM22, PaeI0373, Pfl29ARP and Sma90166) had low positive PC1 values. BpuC26 and BpuT4 defined cluster 4. The only strain belonging to cluster 5 was BsuGB03.

Each cluster was further characterised according to its relative growth-promotion effects on biomass and RSA variables (Fig. 3a and b, respectively). For each variable, the cluster effect was expressed as the mean of the relative differences between the replicates of the strains within a given cluster and the control without bacteria.

### Main volatile compound-mediated modulations of biomass production (Fig. 3a)

**Fig. 4:**
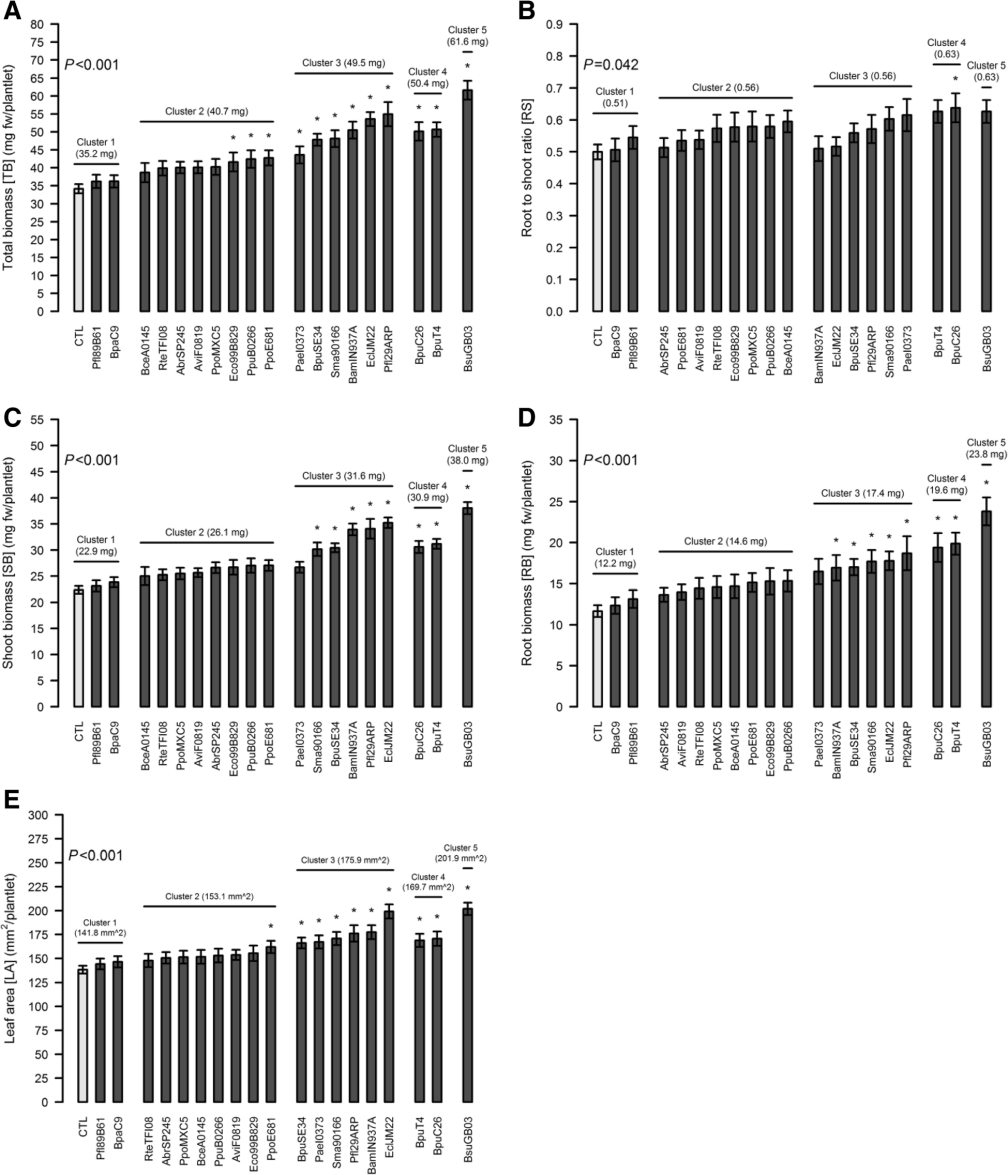
Impact of individual strain volatile compounds on biomass variables. The presented variables are: the TB (**a**), RS (**b**), SB (**c**), RB (**d**) and LA (**e**). The strains are grouped according to the clusters defined earlier, based on PC. Within each cluster, the strains are ranked in ascending mean value order. Presented values are means of the four experimental replicates (64 or 128 biological replicates +/− confidence interval (*α* = 5 %) for each strain and the control, respectively). The *P*-values are displayed on the graphs. Significant changes compared with the control without bacteria are marked with an asterisk (*)

With regard to biomass production, the control plantlets had a TB of 34.1 mg associated with a root-to-shoot ratio (RS) value of 0.50 (Fig. 4a). Relative to the control, the plantlets exposed to the volatiles emitted by the cluster 1 strains showed no significant increase (max +6.2 % for RB) in any of the measured parameters. The overall increase in TB (+19.7 %), due mainly to root growth promotion (+26.8 %), induced by Cluster 2 strains was not significant. The increases in TB observed in clusters 3 and 4 were high (+46.5 and +48 %, respectively), but the plantlets in these clusters differed in terms of biomass allocation. Indeed, Cluster 4 strains induced a higher increase in RB (+69 %). Cluster 5 had the highest increase in biomass production (+80.9 % increase in TB), its RS shift (+27.5 %) being very similar to the cluster 4 value. It is worth noting that the RB production of cluster 5 represented 205.5 % of the control level (+105.5 %, Fig. 3a). Finally, LA was increased by 45.8 % in cluster 5. This trait is proportional to SB production for all the defined clusters.

### Biomass modulation potential of individual strains

Apart from identifying the main grass plant phenotypes modulated by bacterial volatiles exposure, this study also sought to screen individual strains for their growth-promotion ability (Fig. 4).

TB is very significantly modulated by bacterial volatiles (*P* < 0.001). Out of the 19 strains, 12 induced a significant increase in TB production (Fig. 4a), ranging from 41.6 mg (Eco99B829) to 61.6 mg (BsuGB03). As stated earlier, no significant effect was noted for Pfl89B61 and BpaC9 (cluster 1 strains) and this was also the case for all the considered variables.

Only three out of eight cluster 2 strains induced a significant increase in TB production: Eco99B829, PpuB0266 and PpoE681. All three belong to the same sub-group and are characterised by positive PC2 values. All the other biomass-related traits remained unaffected within this cluster, apart from LA for only one strain (PpoE681, Fig. 4e). Due to its narrow PC1 positioning (Fig. 2), cluster 2 showed low intra-cluster variability for biomass production, whatever the variable.

In contrast, cluster 3 strains were more spread out on the PC1 axis and therefore presented greater heterogeneity, with the TB ranging from 43.6 mg (PaeI0373) to 54.9 mg (Pfl29ARP). All six of these strains showed a significant ability to increase TB and LA and only those plantlets exposed to PaeI0373 volatiles did not show any significant changes in SB and RB (Fig. 4c and d, respectively). As observed for cluster 2 strains, individual RS values remained statistically unaffected by all six strains (Fig. 4b).

The cluster 4 strains (BpuC26 and BpuT4) had similar effects on biomass production and both of them increased TB, SB, RB and LA. Compared with clusters 1, 2 and 3, these strains induced higher RB production, leading to a higher mean RS value (0.63), but BpuC26 was the only strain out of all 19 that was able to change RS significantly. Cluster 5′s single *Bacillus subtilis* strain (BsuGB03) induced the highest TB, SB and RB production (61.6 mg, 38.0 mg and 23.8 mg, respectively) without significantly affecting the RS value compared with the control.

### Main volatile-mediated modulations of root system architecture (RSA)

**Fig. 5:**
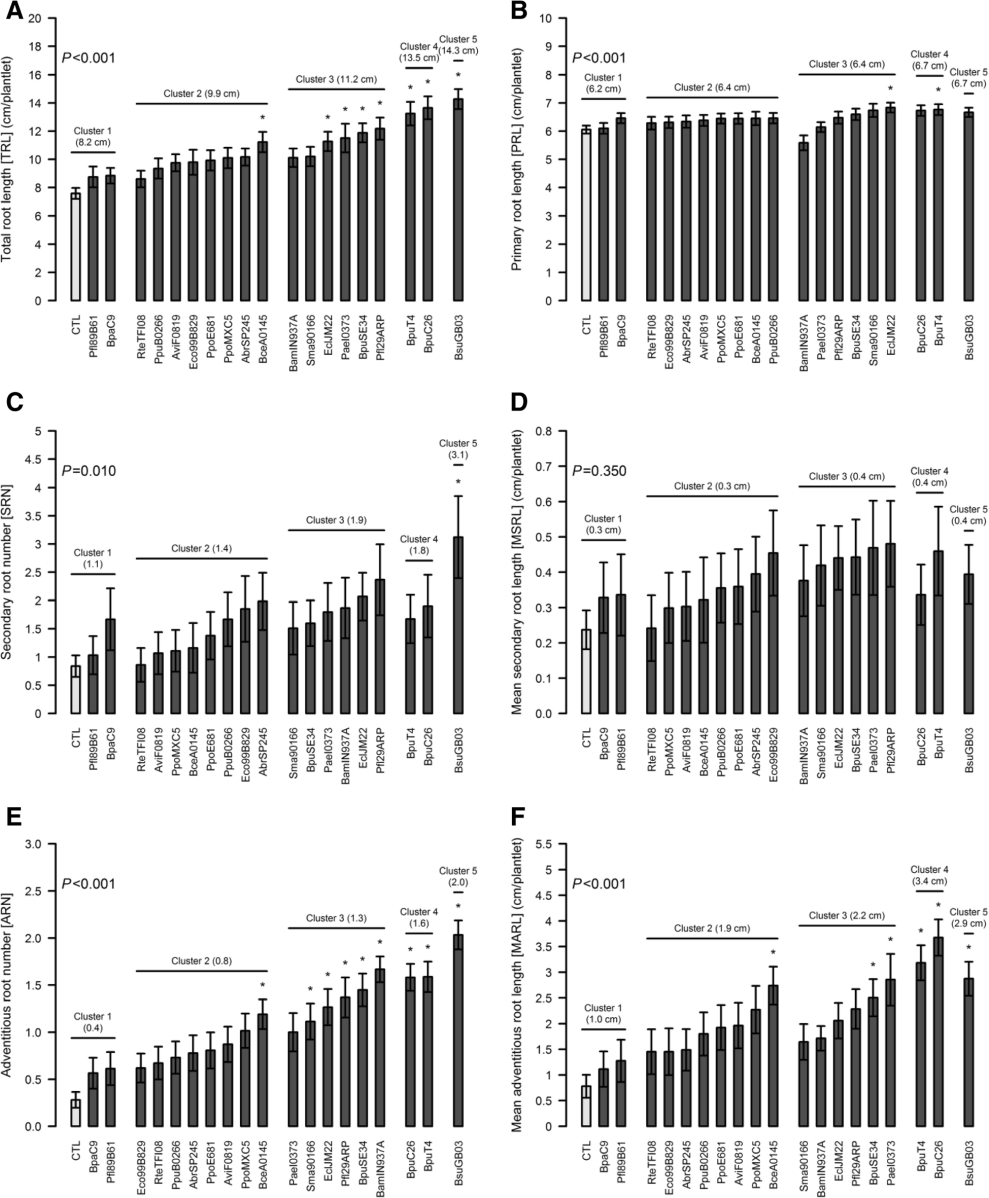
Impact of individual strain volatile compounds on the main RSA variables. The presented variables are the TRL (**a**), PRL (**b**), SRN (**c**), MSRL (**d**), ARN (**e**) and MARL (**f**). The strains are grouped according to the clusters defined earlier, based on PC. Within each cluster, the strains are ranked in ascending mean value order. Presented values are means of the four experimental replicates (64 or 128 biological replicates +/− confidence interval (*α* = 5 %) for each strain and the control, respectively). The *P*-values are displayed on the graphs. Significant changes compared with the control without bacteria are marked with an asterisk (*)

With regard to RSA, the most correlated variables to PC1 and PC2 were selected to characterise each strain group (Fig. 3b). The control plantlets presented a TRL of 7.6 cm, with limited secondary (*SRN* = 0.8; *TSRL* = 0.5 cm) or adventitious root production (*TARL* = 1 cm). SRD was therefore limited to 0.1 secondary root cm^−1^ of primary root (Fig. 5, Additional file 1: Figure S1). Cluster 1 showed no significant increase either in biomass production or RSA parameters. Unlike biomass production, Cluster 2 strains were able to induce a 30.8 % overall increase in TRL. The RSA modulation ability of clusters 3 and 4 was consistent with their respective RS values. Both clusters greatly promoted total biomass production, but the TRL increase in cluster 3 was limited to 48.4 %, compared with a 78.7 % increase in cluster 4. The TARL increase was higher in cluster 4 (+441.5 %) than in cluster 3 (+229.7 %). Cluster 5 had the highest increase in TRL (+88.5 %), due almost entirely to increases in TARL (+474.5 %) and SRN (+293 %) compared with the control without bacteria. The MSRL increase (+65.9 %) was not significant.

### Modulation of the root system architecture (RSA) by individual strains

Overall, the variability in the RSA parameters was higher than that of the biomass variables, apart from TRL and primary root length (PRL, Fig. 5). None of the cluster 1 strains induced significant changes in RSA variables.

With regard to cluster 2 strains, only BceA0145 induced significant changes in TRL, ARN and MARL (Fig. 5a, e and f). Therefore, it is mostly responsible for the aforementioned overall cluster 2 significant change in TRL. None of these strains affected PRL, SRN or MSRL significantly (Fig. 5b, c and d). It should be noted that PC2-positive strains showed the highest SRN and MSRL within this cluster. Apart from RteTFI08, the same was true for PC2-negative ones with regard to ARN and MARL. Four out of six strains increased TRL in cluster 3. In contrast, BamIN937A and Sma90166 did not induce significant change in TRL and gave the lowest MSRL and MARL values. In addition, BamIN937A volatiles seemed to slightly reduce PRL (5.6 cm) compared with the control (6.1 cm). This negative effect of BamIN937A volatiles on PRL was balanced by an average SRN (1.9) and a high ARN (1.7). The only cluster 3 strain that increased PRL was EclJM22 (6.8 cm). At the cluster 3 level, no statistically significant effect was measured for SRN and MSRL. All cluster 3 strains increased ARN, apart from PaeI0373. This strain, together with BpuSE34, induced the production of significantly longer adventitious roots (Fig. 5f). Both ARN and MARL showed high intra-cluster variability, ranging from 1.0 to 1.7 and from 1.6 to 2.9 cm plantlet^−1^, respectively.

Cluster 4 strains increased TRL, ARN and MARL, but had no significant effect on SRN and MSRL. On average, contrary to secondary root traits, cluster 4 strains promoted adventitious root growth more effectively than cluster 3 strains did. Only BpuT4 significantly enhanced PRL (6.8 cm). As illustrated in Fig. 5b, this RSA parameter was one of the traits least affected by bacterial volatiles.

The cluster 5 strain (BsuGB03) had a significant impact on most RSA parameters, apart from PRL and MSRL. It induced the highest TRL (14.3 cm), explained mainly by high SRN and ARN values (3.1 and 2.0, respectively, *vs* 0.8 and 0.3, respectively, for the control without bacteria).

### Correlations between biomass production and root system architecture (RSA) traits

In our experiment, TB production was correlated mainly with TRL, SRN, ARN and SRD, with *r* values ranging from 0.82 to 0.89, and to a lesser extent with TSRL and TARL, with *r* values of 0.72 and 0.75, respectively. PRL was the RSA parameter least correlated (*r* = 0.41) with TB and it was not correlated with other RSA parameters either positively (generalised root growth promotion) or negatively (compensatory effect between primary root and secondary or adventitious root growth).

## Discussion

### Bacterial volatiles have a significant impact on the early developmental stages of a model grass

As shown in Fig. 1, representative plantlets subjected to BsuGB03 volatiles reached the 3-leaf stage (stage 13, [40]) after 10 days of co-cultivation, whereas control plantlets had only two unfolded leaves (stage 12). This observation is consistent with the results reported by [41], indicating that PGPR can induce significant changes in plant growth rate. This could also explain the observed RSA differences because *B. distachyon* plantlets are known to produce up to two coleoptile nodal adventitious roots at stage 13 [39]. In our *in* vitro system, TB production was strongly correlated with traits related to secondary and adventitious root growth. The correlation between TB production and PRL was weaker. Similar correlation results between TB and PRL, as well as TSRL, were observed for *A. thaliana* by [7], indicating that a branched root system phenotype seems to be associated with increased SB production.

Apart from BpuC26, the biomass allocation (RS) of the plantlets was not significantly influenced by bacterial volatiles. The observed growth-promotion effects therefore did not seem to be due to energy being used to increase root growth instead of shoot development.

### Contrasting biomass and root system architecture (RSA) modulations define the five groups of bacterial strains

The bacterial volatiles used in this study led to five groups of phenotypes being defined.

Group 1 strains (BpaC9 and Pfl89B61) did not cause any significant change after 10 days in either plant biomass production or RSA.

Three (Eco99B829, PpuB0266 and PpoE681) out of eight strains in Group 2 were able to increase plant total biomass significantly. This reflected altered root branching characterised by a higher SRN and MSRL.

Group 3 was characterised by high biomass production, but moderate impact on TRL. This group contained BamIN937A, BpuSE34, EclJM22, PaeI0373, Pfl29ARP and Sma90166, which were all able to promote plant growth significantly.

Group 4 contained two strains belonging to the same species and showing high growth-promotion potential: BpuC26 and BpuT4. They both induced a great increase in RB and TRL, which significantly affected biomass allocation for BpuC26.

The single strain in cluster 5, BsuGB03, showed the highest biomass production, with RB representing 205.5 % of the control level. BsuGB03 induced the highest increase in TRL (+88.5 %), due almost entirely to increases in TARL (+474.5 %) and SRN (+293 %), without significantly affecting the growth of the primary root.

The plant growth modulation abilities of BsuGB03, BamIN937A, EclJM22, BpaC9, Pfl89B61 and *Burkholderia cepacia* were consistent with those observed by [6] and [11] on *A. thaliana*. The volatiles emitted by Sma90166, BpuT4, Eco99B829, *Pseudomonas aeruginosa* and *Pseudomonas putida*, however, improved *B. distachyon* biomass production without having any significant effect on *A. thaliana* growth [6, 11]. The observed differences could be due to the receiving plant species or to technical constraints (e.g., bacterial and plant cultivation medium composition, Petri dish volume or inclination angle), resulting in different volatile concentrations and perceptions in the sealed system.

### Variability exists up to the intra-specific level and is not related to taxonomy

Our results accord with the existing literature in that they suggest that the growth-promoting effect of particular strains is specific [7, 41]. With the bacterial volatile emission profiles differing at the genus, species and strain levels [11, 42], it is likely that their volatile-mediated growth-promotion ability will vary. Strains belonging to the same species can induce fairly consistent plant growth promotion (e.g., *Bacillus pumilus* and *Paenibacillus polymyxa* strains) or have more variable effects on biomass production and RSA (e.g., *Pseudomonas fluorescens* strains).

Previous research on PGPR focused mainly on Gram-negative strains [7, 43, 44]. More recently, *Bacillus* strains have been tested for their growth-promoting ability [7]. In the present study, our strain selection included both Gram-positive and Gram-negative bacteria (Table 1). From a physiological point of view, most Gram-negative bacteria are unable to form spores [45]. This could affect their survival rate under natural adverse conditions or during formulation or storage prior to application [43]. Under our screening conditions, growth promotion was observed for both Gram-positive and Gram-negative strains, which is consistent with the results obtained on *A. thaliana* for BsuGB03 and EclJM22 [6]. Although cluster 4 and 5 strains belonged to the Gram-positive *Bacillus* genus, no clear trend appeared that would support the hypothesis that Gram-positive strains possess higher growth promotion ability. This point was emphasised by: (1) the cluster 3 and 2 strain composition; (2) the presence of a *Bacillus* strain (BpaC9) in the negative control cluster; and (3) the polyphyletic nature of bacterial groups based on Gram staining results [46, 47].

**Table 1:** Characteristics of the bacterial strains used in the study. For each of the 19 strains, the acronym, Gram type, family, reported ecophysiological characteristics and bibliographical references are presented

Strain	Acronym	Gram type	Family	Characteristics and references
*Azospirillum brasilense* SP245	AbrSP245	-	*Rhodospirillaceae*	Associative microaerophilic diazotroph [63]
*Azotobacter vinelandii* A60 - F08 19	AviF0819	-	*Pseudomonadaceae*	Free-living aerobic diazotroph [64]
*Bacillus amyloliquefaciens* AP278 - IN937a	BamIN937a	+	*Bacillaceae*	Some strains are diazotrophic or facultative microaerophilic; many *Bacillus* produce antibiotics ([4, 6, 15, 27, 65–67], *newly isolated strain)
*Bacillus pasteurii* AP277 - C9	BpaC9	+	*Bacillaceae*	
*Bacillus pumilus* AP280 - T4	BpuT4	+	*Bacillaceae*	
*Bacillus pumilus* AP281 - SE34	BpuSE34	+	*Bacillaceae*	
*Bacillus pumilus* C26*	BpuC26	+	*Bacillaceae*	
*Bacillus subtilis* AP305 - GB03	BsuGB03	+	*Bacillaceae*	
*Burkholderia cepacia* A01-45	BceA0145	-	*Burkholderiaceae*	Rarely diazotrophic, associative endophytic nitrogen fixer, wheat PGPR [68]
*Enterobacter cloacae* AP12 - JM22	EclJM22	-	*Enterobacteriaceae*	PGPR [6]
*Escherichia coli* DH5α 99B829	Eco99B829	-	*Enterobacteriaceae*	Bacterial control [6]
*Paenibacillus polymyxa* AP294 - E681	PpoE681	+	*Paenibacillaceae*	Facultative microaerophilic, can produce phytohormones analogs, suppress pathogens and solubilize organic phosphate ([4, 27], *newly isolated strain)
*Paenibacillus polymyxa* MXC5*	PpoMXC5	+	*Paenibacillaceae*	
*Pseudomonas aeruginosa* I03-73	PaeI0373	-	*Pseudomonadaceae*	Associative wheat PGPR [68]
*Pseudomonas fluorescens* AP2 - 89B61	Pfl89B61	-	*Pseudomonadaceae*	
*Pseudomonas fluorescens* Pf29Arp	Pfl29ARP	-	*Pseudomonadaceae*	
*Pseudomonas putida* KT2440 - B02 66	PpuB0266	-	*Pseudomonadaceae*	
*Raoultella terrigena* Tfi08*	RteTFI08	-	*Enterobacteriaceae*	Aerobic or facultatively anaerobic, *newly isolated
*Serratia marcescens* AP4 - 90 166	Sma90166	-	*Enterobacteriaceae*	PGPR [4, 6, 27]

### Contrasting effects indicate some heterogeneity in bacterial volatile production

The induced changes in the plantlet phenotypes varied greatly from one cluster to another. We hypothesize, therefore, that the volatile blends emitted by the bacteria were in line with this observation, both quantitatively (volatile concentrations) and qualitatively (volatile identities). This hypothesis has to be assessed based on a thorough analysis of the volatile emission kinetics of the strains used in the present study. Among the putative bioactive volatiles, the most important and prominent inorganic volatiles released by bacteria are ammonia (especially on a protein-rich medium), carbon dioxide and HCN. Moreover, 2,3-butanediol and its precursor, acetoin, are likely to be produced on the sucrose-containing, low pH Murashige & Skoog medium [8, 15]. Microbes simultaneously produce 2,3-butanediol and CO_2_ from pyruvate by a fermentation process that involves the synthesis of the volatile precursor, 3-hydroxy-2-butanone (acetoin) [30]. *In vitro*, the observed volatile-mediated growth-promotion effects could therefore be at least partially linked to CO_2_ emissions [13, 48]. A significant increase in CO_2_ concentration due to bacterial emission, however, was unlikely in our experiment because it was sealed by CO_2_-permeable Parafilm ® [11]. Experiments testing this hypothesis could be performed by either absorbing the CO_2_ with Ba(OH)_2_, measuring CO_2_ in the reaction vessel or performing the experiments without any seal. With regard to the other biologically-active bacterial volatiles [8, 16–19], their roles in the growth promotion of *B. distachyon* remain to be investigated. More specifically, the volatile impacts on targeted processes driving the primary, secondary (lateral) and adventitious (crown) root development should be studied. Indeed, it is well known that bacterial volatiles are able to modulate the main hormonal pathways [4, 6, 20, 21] and that both specific and shared hormonal pathways are involved in postembryonic root development [49–51].

### From *in vitro* conditions to the field

The *in vitro* co-cultivation system used in this study enabled us to screen 19 bacterial strains for their volatile-mediated plant growth-promotion ability over a 10-day co-cultivation period. The *B. distachyon* plantlets were grown on near-vertical agar plates alongside a bacterial inoculum developed on a Murashige & Skoog medium that was supplemented with sucrose and tryptic soy agar [15]. Previous studies have demonstrated that VOC profiles and the growth promotion effects of bacterial volatiles could depend on inoculum size and cultivation medium composition [11]. Due to technical constraints related to the overnight growth of the 19 bacterial suspensions, our inoculum size was limited to 2*10^6^ colony-forming units (20 μl of a 10^8^ colony-forming units mL^−1^ cell suspension), which is lower than the values used by [6] and [7]. The medium [15] used in this study was a Murashige & Skoog-based medium that allowed all 19 strains to grow, while emitting a limited amount of VOCs itself (Farag M., pers. com.). It is worth noting that the inoculum dose did not influence results when nutrient-poor media such as Murashige & Skoog or Angle media were used in other studies [11]. In addition, plant growth-promotion effects mediated by *Bacillus* volatiles were observed only when a Murashige & Skoog medium was used to grow the bacteria as well as the plants [8]. So the observed effects of different bacterial volatiles on plant growth need to be qualified as being media dependent. The same holds true for the results of the present study.

Transposing obtained results to field conditions is not straightforward and remains challenging [52]. This is due partly to the technical constraints (*e.g.,* Petri dish size and volume, near-vertical cocultivation system) that limit the duration of the plant cultivation process, and thus restricted the scope of the present study to early developmental stages. In the field, in order to optimize resource-use efficiency under agronomical conditions, the main root foraging zones should ideally be located in the resource-rich areas of the soil [53]. In the present study, the observed RSA modulations could have contributed to greater tolerance of transient or prolonged drought stress because of increased root branching in the soil upper layers (topsoil foraging) or, to a lesser extent, a longer primary root (deep soil foraging), respectively. Such root phenotypes could also help plants efficiently acquire either relatively immobile (P, K) or mobile (N) nutrients [53–55]. Moreover, various constraints (*e.g.,* endogenous soil microbial populations, soil composition, porosity and aeration, root rhizodeposition, etc.) may influence the bacterial growth and release of volatiles, hence modulating the outcome of the plant-bacteria interactions mediated by volatile compounds under field conditions.

### Future prospects

The volatile emission kinetics of the complete set of strains are now being characterised in order to identify volatile compounds/mixtures that promote *B. distachyon* growth. There are more than 770 bacterial VOCs in the SuperScent public database (http://bioinf-applied.charite.de/superscent/index.php?site1/4home, [9, 13]) and about 1000 microbial VOCs from 350 bacterial and 80 fungal species in the ‘mVOC’ database (http://bioinformatics.charite.de/mvoc/, [5, 56]). Once the active compounds have been identified, experiments closer to field conditions should be performed. On the one hand, *B. distachyon* plantlets could be exposed to the bacterial volatile blends based on an *ex vitro* set-up similar to the one designed by [48] or [19]. This would allow the plants to (1) perceive the volatile signature in the root system without directly exposing the whole plant to the volatile blend and (2) reach older developmental stages corresponding to a mature RSA. On the other hand, slow-release formulations of the VOC candidates like those used in integrated pest management [57] could be used to expose the root system to controlled VOC concentrations and assess their effects in real soil conditions. Indeed, the VOC diffusion rate from their release point could be influenced by their polarity and the physico-chemical characteristics of the soil matrix [12, 13].

## Conclusions

To the best of our knowledge, this study is the first report on bacterial volatile-mediated growth promotion of a grass plant. Using an *in vitro* near-vertical screening set-up, five groups of strains inducing characteristic changes in a *B. distachyon* phenotype were defined. Contrasting modulations of biomass production coupled with changes in RSA were observed. Most of the strains that increased total plant biomass also modulated adventitious root growth. Under our screening conditions, total biomass production was strongly correlated with the length and branching of the root system components, except for primary root length. Irrespective of the considered phenotypic variables, *B. subtilis* GB03’s volatile compounds induced the most significant changes. Considering the great diversity of bacterial volatile production, further experiments are needed to characterise the inorganic and organic volatile emission kinetics of these bacterial strains in order to identify the candidates responsible for the observed growth-promotion effects and to assess their influence on plant growth under agricultural soil conditions.

## Methods

### Plant material

*Brachypodium distachyon* (line Bd21) caryopses were kindly provided by Dr Philippe Vain from the John Innes Centre (Norwich, UK) and propagated under greenhouse conditions in 2009.

### Bacterial strains

The bacterial strains used in the present study were selected based on their potential PGPR properties and/or their role in the nitrogen cycle (Table 1). *Bacillus amyloliquefaciens* In937A (BamIN937A), *Bacillus pasteurii* C9 (BpaC9), *Bacillus pumilus* T4 (BpuT4), *Bacillus pumilus* SE34 (BpuSE34), *Bacillus subtilis* GB03 (BsuGB03), *Enterobacter cloacae* JM22 (EclJM22), *Escherichia coli* DH5α 99B829 (Eco99B829), *Paenibacillus polymyxa* E681 (PpoE681), *Pseudomonas fluorescens* 89B61 (Pfl89B61) and *Serratia marcescens* 90166 (Sma90166) were kindly provided by Dr Paul W. Paré and Dr John McInroy (Texas Tech University, Lubbock, TX, USA). *Pseudomonas fluorescens* 29ARP (Pfl29ARP) was kindly provided by Dr Alain Sarniguet (Institut National de la Recherche Agronomique, Rennes, France). *Azospirillum brasilense* SP245 (AbrSP245), *Azotobacter vinelandii* F0819 *(*AviF0819), *Bacillus pumilus* C26 (BpuC26), *Burkholderia cepacia* A01-45 (BceA0145), *Paenibacillus polymyxa* MXC5 (PpoMXC5), *Pseudomonas aeruginosa* I03-73 (PaeI0373), *Pseudomonas putida* KT2440-B0266 (PpuB0266) and *Raoultella terrigena* Tfi08 (RteTFI08) came from the Katholieke Universiteit Leuven collection (Leuven, Belgium).

The main characteristics of these bacterial strains, including acronyms used in the study, Gram type and taxonomic position, are presented in Table 1.

### *In vitro* screening

*Brachypodium distachyon* plantlets and bacterial strains were co-cultivated *in vitro* for 10 days at 22 °C in a system combining a 12-cm square Petri dish (plant compartment) with the bottom part of a 3.5-cm round one (bacterial compartment) in order to allow interactions throughout the gaseous phase only (Fig. 1).

The caryopses were surface-sterilised, as described by [58] and [59]. After 2 h of incubation in distilled water at room temperature, lemmas were removed and the grains were stored in distilled water. They were then successively incubated for 30 s in 70 % v/v ethanol, rinsed once in sterile distilled water and surface-sterilised for 4 min in 1.4 % v/v sodium hypochlorite solution under manual agitation, before being washed three times with sterile distilled water. The caryopses were stratified in the dark for 2 days at 4 °C on 0.8 % w/v plant agar (Duchefa Biochemie B.V., Haarlem, The Netherlands) plates containing 1× Hoagland’s medium (6 mM KNO_3_, 4 mM Ca(NO_3_)_2_, 2 mM MgSO_4_, 1 mM NH_4_H_2_PO_4_, 17.97 μM ferric tartrate.2H_2_O, 46.25 μM H_3_BO_3_, 9.15 μM MnCl_2_.4H_2_O, 0.77 μM ZnSO_4_.7H_2_O, 0.32 μM CuSO_4_.5H_2_O, 0.11 μM NaMoO_4_.2H_2_O, Ref. 30630037–1, Plantmedia, Dublin, OH, USA), before being transferred for 24 h at 22 °C under 94 μmol m^−2^ s^−1^ in the PAR (LED lighting) with a 20-h photoperiod, in line with the BrachyTAG culture protocol [58, 59]. The caryopses were positioned on top of the agar plate, the embryo facing the Petri dish cover. The dishes were inclined at a 65° angle to ensure proper root growth on top of the agar plates [7].

One week before the onset of the co-cultivation, each bacterial strain was freshly plated from its 20 % v/v glycerol stock suspension stored at −80 °C. All the aforementioned strains were plated on 4 % w/v tryptic soy agar (Sigma-Aldrich, Saint-Louis, MO, USA), apart from (1) Pfl29ARP and AviF0819, which were plated on 2.5 % w/v lysogeny broth (Sigma-Aldrich) containing 1.5 % w/v agar for microbiology (Sigma-Aldrich) and (2) AbrSP245 and BceA0145, which were grown on modified lysogeny broth agar supplemented with 2.5 mM CaCl_2_ and 2.5 mM MgSO_4_. The strains were plated again on their corresponding media 24 h before starting the overnight cultures in liquid medium ([15] 1× Murashige & Skoog medium [Duchefa Biochemie B.V.], complemented with 1.5 % sucrose and 0.4 % tryptic soy broth [Sigma-Aldrich]). Each strain concentration was subsequently adjusted to 10^8^ colony-forming units mL^−1^ before the start of the co-cultivation step.

After 24 h of germination at 22 °C, two *B. distachyon* plantlets were transferred to each square 12-cm Petri dish containing 45 mL of Hoagland’s medium (Plantmedia). Then 20 μL of each bacteria suspension were pipetted in the bacterial compartment containing 3 mL of [15] medium (1× Murashige & Skoog medium supplemented with 1.5 % w/v agar for microbiology [Sigma-Aldrich], 1.5 % sucrose and 0.4 % tryptic soy agar [Sigma-Aldrich]). The bacterial suspension droplet was dried under a laminar flow before the 12-cm Petri dish was sealed with Parafilm® (Pechiney Plastic Packaging Company, Chicago, IL, USA; [6]). The resulting co-cultivation dishes allowed the growth of the plantlets over 10 days in the presence of the bacteria at 22 °C under 94 μmol m^−2^ s^−1^ in the PAR (LED lighting) with a 20-h photoperiod. Four independent experimental replicates were performed. Within each experiment, 16 plantlets were considered for each strain, and 32 plantlets were used for the control without bacteria that contained only the [15] medium. At the end of the co-cultivation process, TB, SB, RB, RS, LA and nine RSA parameters were recorded.

### Leaf area (LA) measurements

After 10 days of co-cultivation, the Petri dishes were opened to get rid of the condensation water and prepare the roots and leaves for data collection. Each Petri dish was photographed with a 10 megapixel Finepix HS10 camera (Fujifilm Holdings, Tokyo, Japan). Projected LA was measured for each plantlet using MVHimage software v8 (Global Systems Science, Boston, MA, USA), in line with the manufacturer’s instructions.

### Root system architecture (RSA) analysis

Each root system was manually untangled with a needle in order to separate intermingled lateral roots before scanning at 200 dpi on an HP Scanjet G4010 A4 scanner (Hewlett-Packard, Palo Alto, CA, USA). The resulting images were analysed with EZ-Rhizo software [60], in line with the manufacturer’s instructions. Each output file was manually checked and edited in order to correct errors in the automatic detection of the roots. Secondary and adventitious roots smaller than 1 mm were discarded and not taken into account for the calculation of RSA parameters and multivariate analysis. For each plantlet, PRL, SRN, MSRL, TSRL, SRD (number of secondary roots per cm of primary root), ARN, MARL, TARL and TRL were recorded.

### Statistical analysis

Multivariate and univariate statistical analysis were performed with R 3.0.3 software [61] running the FactoMineR package 1.25 [62]. Using the 14 measured variables, PCA was performed on data that were weighted, centred and scaled to zero mean and unit variance. This approach assigned the same total weight for biomass and RSA variable classes and limited the bias associated with the number of biomass-related variables compared with the number of recorded RSA parameters (5 *vs* 9, respectively). Within each class of variables, each measured parameter had the same weight irrespective of its order of magnitude. The resulting 14 PCs were then used as input variables for hierarchical clustering based on the Euclidian distance and the Ward algorithm.

After verifying the overall application conditions based on Normal Q-Q (normality check) and Scale Location (equality of variance check) plots, each variable was subjected to a two-way analysis of variance (ANOVA) using the strains as the fixed factor, the four experimental replicates as the random factor and their interaction as the basis of comparison. When strain influence on a particular variable was significant (p < 0.05), means were separated using Dunnett’s test. In the figures, mean values that were significantly distinct from the control (*α* = 5 %) are marked with an asterisk (*).

## Availability of supporting data

The data set supporting the results of this article is included within the article and its additional file.

## Additional file

Additional file 1: Figure S1. Impact of individual strain volatile compounds on Total Secondary Root Length (A), Total Adventitious Root Length (B) and Secondary Root Density (C). The strains are grouped according to the clusters defined earlier, based on PC. Within each cluster, the strains are ranked in ascending mean value order. Presented values are means of the four experimental replicates (64 or 128 biological replicates +/− confidence interval (*α* = 5 %) for each strain and the control, respectively). Significant changes compared with the control without bacteria are marked with an asterisk (*). (DOCX 374 kb)

## Abbreviations

ANOVA: Analysis of variance
AR: Adventitious root
ARN: Adventitious root number
LA: Leaf area
MARL: Mean adventitious root length
MSRL: Mean secondary root length
PC: Principal component
PCA: Principal component analysis
PGPR: Plant growth-promoting rhizobacteria
PR: Primary root
PRL: Primary root length
RB: Root biomass
RS: Root-to-shoot ratio
RSA: Root system architecture
SB: Shoot biomass
SR: Secondary root
SRD: Secondary root density
SRN: Secondary root number
TARL: Total adventitious root length
TB: Total biomass
TRL: Total root length
TSRL: Total secondary root length
VOC: Volatile organic compound
