# Supplementary material for: Influence of rhizobacterial volatiles on the root system architecture and the production and allocation of biomass in the model grass Brachypodium distachyon (L.) P. Beauv
Source: BMC Plant Biol. 2015 Aug 12;15:195. doi: 10.1186/s12870-015-0585-3 (PMC4531529; doi:10.1186/s12870-015-0585-3)

BMC Plant Biology

Influence of rhizobacterial volatiles on the root system architecture and the production and allocation of biomass in the model grass *Brachypodium distachyon* (L.) P. Beauv.

Pierre Delaplace, Benjamin M. Delory, Caroline Baudson, Magdalena Mendaluk-Saunier de Cazenave, Stijn Spaepen, Sébastien Varin, Yves Brostaux, Patrick du Jardin.

**Fig. S1. Impact of individual strain volatile compounds on Total Secondary Root Length (A), Total Adventitious Root Length (B) and Secondary Root Density (C).** The strains are grouped according to the clusters defined earlier, based on PC. Within each cluster, the strains are ranked in ascending mean value order. Presented values are means of the four experimental replicates (64 or 128 biological replicates +/- confidence interval (α = 5%) for each strain and the control, respectively). Significant changes compared with the control without bacteria are marked with an asterisk (*).


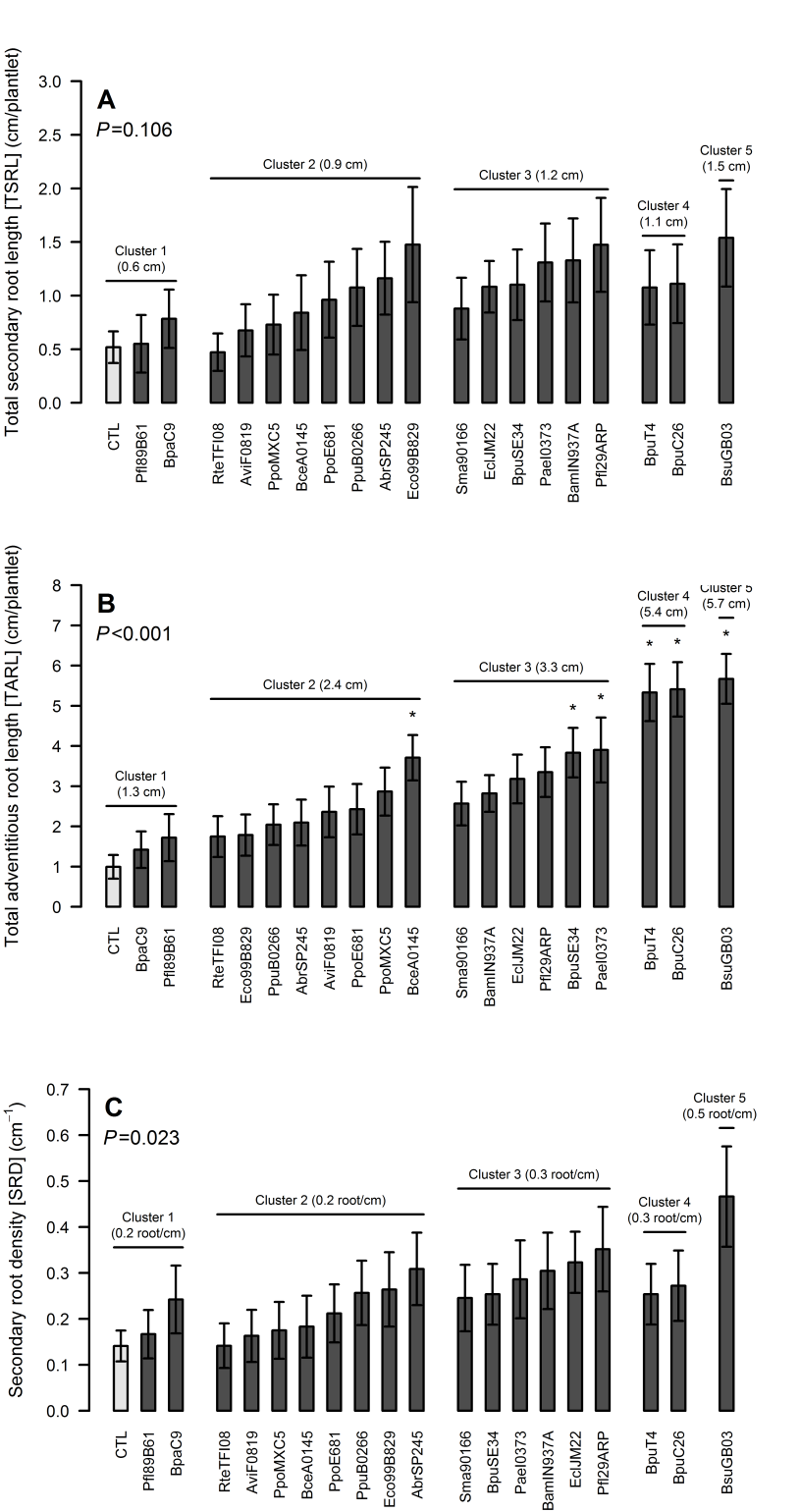

Supplement: Additional file 1: Figure S1. — Impact of individual strain volatile compounds on Total Secondary Root Length (A), Total Adventitious Root Length (B) and Secondary Root Density (C). The strains are grouped according to the clusters defined earlier, based on PC. Within each cluster, the strains are ranked in ascending mean value order. Presented values are means of the four experimental replicates (64 or 128 biological replicates +/− confidence interval (α = 5 %) for each strain and the control, respectively). Significant changes compared with the control without bacteria are marked with an asterisk (*). (DOCX 374 kb) [file 12870_2015_585_MOESM1_ESM.docx]
